# Comparison of endoscopic breast-conserving surgery versus conventional breast-conserving surgery for the treatment of early-stage breast cancer: a meta-analysis

**DOI:** 10.3389/fonc.2024.1419123

**Published:** 2024-08-06

**Authors:** Liwen Li, Yiwen Liang, Chunyan Li, Miaoyan Huang, Weiming Liang, Tian Qin

**Affiliations:** The First Affiliated Hospital of Guangxi University of Science and Technology, Guangxi University of Science and Technology, Liuzhou, China

**Keywords:** breast cancer, breast-conserving surgery, endoscopic, recurrence, meta-analysis

## Abstract

**Introduction:**

This meta-analysis seeks to evaluate the efficacy and safety of endoscopic breast-conserving surgery (E-BCS) compared to conventional breast cancer surgery (C-BCS) in patients diagnosed with early-stage breast cancer.

**Materials and methods:**

Four databases (Medline, Embase, Web of Science and CENTRAL) were searched published from establishment of database to January 30,2024, for articles studying E-BCS compared to C-BCS in patients diagnosed with early-stage breast cancer. Meta-analyses of procedure time, blood loss, length of incision, drainage duration, total postoperative drainage volume, average duration of hospital stay, positive rate of margin, complication rate, recurrence rate, metastasis rate and cosmetic scoring were performed.

**Results:**

Totally 11 studies were included for meta-analysis. Compared with C-BCS, E-BCS exhibited significantly reduced incision length (WMD = -6.44, 95%CI: -10.78 to -2.11, P=0.004, I^2^ = 99.0%) and superior cosmetic scoring (WMD = 2.69, 95%CI: 1.46 to 3.93, P=0.001, I^2^ = 93.2%), but had significantly longer operation time (WMD = 34.22, 95%CI: 20.89~47.55, P=0.000, I^2^ = 90.7%) and blood loss (WMD = 3.65, 95%CI: -3.12 to 10.43, P=0.291, I^2^ = 86.8%). There was no significant difference in terms of recurrence rate, metastasis rate, positive rate of tumor resection margins, drainage duration, drainage volume, complication rate and hospital days.

**Conclusions:**

Our research findings indicate that E-BCS is a viable and secure method for treating breast cancer in its early stages. E-BCS provides distinct advantages in terms of the length of the incision and the aesthetic result, without demonstrating an elevated recurrence rate or metastasis rate.

**Systematic review registration:**

https://www.crd.york.ac.uk/prospero/display_record.php?ID=CRD42024535164, identifier CRD42024535164.

## 1 Introduction

Breast cancer is characterized by the excessive and unregulated proliferation of breast cells, resulting in the formation of tumors, and women bear a significant burden of this disease (1, 2). As of 2020, female breast cancer has overtaken lung cancer to become the most prevalent form of cancer globally, accounting for 11.7% of all cases. Additionally, it is now the fifth greatest cause of cancer-related deaths worldwide, responsible for 6.9% of all fatalities (3, 4). Patients diagnosed with early-stage breast cancer often have breast-conserving surgery (BCS) followed by adjuvant radiation therapy. This approach has been proven to be a viable and successful alternative to total mastectomy, and extensive research has demonstrated its safety (5–7). Nevertheless, conventional breast-conserving surgery (C-BCS) continues to have certain drawbacks in terms of its impact on breast aesthetics (8–10). From a clinical standpoint, there are still some patients who are not fully satisfied with the aesthetic outcome of breast-conserving surgery (11). To address the physiological and psychological needs of breast cancer patients and enhance patient satisfaction, clinical surgical treatment has been focused on achieving minimally invasive and aesthetically pleasing operations while ensuring safety. This has led to the advancement of new techniques for breast-conserving surgery, such as endoscopic breast-conserving surgery (E-BCS) (12).

During the 19th century, Dr. Desormeaux, a French physician, invented the first endoscopic technique for the examination of the urinary system and bladder, and also introduced this technique in the field of gynaecology (13, 14). As science and technology continue to advance, there is a growing number of therapeutic therapies that integrate endoscopic technology and microsurgery (15–17). Currently, endoscopic technology has emerged as a prominent technique in the realm of minimally invasive surgery and is extensively employed in breast surgery. This precise and minimally invasive approach preserves the functionality of the surgery area while significantly enhancing the aesthetic appeal of the breasts (18–20). Studies has shown that endoscopic assisted breast-conserving surgery offers a cosmetic benefit, leading to increased patient satisfaction and enhanced postoperative quality of life (21–26). Nevertheless, endoscopic technology poses challenges, and endoscopic breast surgery involves a sequence of specialized surgical techniques. There is a potential risk of tumor diffusion, and the safety of this operation in relation to tumor management lacks adequate supporting evidence (10). Hence, the application of E-BCS in the management of early breast cancer remains a subject of debate, necessitating additional validation of its safety and dependability.

Therefore, we conducted a meta-analysis to compare the effectiveness and safety of E-BCS versus C-BCS in patients with early-stage breast cancer.

## 2 Materials and methods

### 2.1 Search strategy

This meta-analysis adhered to the 2020 principles set forth by the Preferred Reporting Project for Systematic Review and Meta-Analysis (PRISMA). The study has been officially registered at PROSPERO under the registration number CRD42024529976. A thorough search was conducted in four databases, namely PubMed, Embase, Web of Science, and the Cochrane Library, to gather literature published until January 30, 2024. The search methodology followed the PICOS principle and employed a combination of MeSH terms and unrestricted text phrases. The search approach utilized involved combining the terms “Breast Cancer”, “endoscopic”, and “breast-conserving surgery”. **Supplementary Material 1** provided a comprehensive overview of the search record.

### 2.2 Inclusion and exclusion criteria

Inclusion criteria were as follows (1) patients diagnosed as early-stage breast cancer; (2) patients in the intervention group received E-BCS; (3) patients in the control group received C-BCS; (4) at least one of the following outcomes were reported: operation time, intraoperative bleeding volume, incision length, postoperative drainage time, total postoperative drainage rate, complications, recurrence rate, positive tumor resection margin rate, hospital days, and cosmetic effect; (5) study design: randomized controlled trial, prospective study, and retrospective study.

The exclusion criteria are as follows: (1) other types of articles, such as case reports, protocols, letters, editorials, comments, reviews, meta-analyses; (2) Non breast cancer; (3) not E-BCS versus C-BCS; (4) duplicate patient cohort; (5) data cannot be extracted.

### 2.3 Selection of studies

The process of literature selection, which involved removing duplicate entries, was conducted using EndNote (Version 20; Clarivate Analytics). Two autonomous reviewers carried out the initial search. The duplicate entries were eliminated, and the titles and abstracts were assessed to establish their relevancy. Each study was then categorized as either included or excluded. We resolved the issue by reaching a consensus. If the parties involved cannot reach an agreement, a third reviewer takes on the role of a mediator.

### 2.4 Data extraction

Two independent reviewers extracted data. The extracted data included: (1) Basic characteristics of studies included: author, nationality, year of publication; (2) Baseline characteristics of study subjects: age, sample size, tumor stage; (3) outcome indicators: operation time, intraoperative blood loss, incision length, postoperative drainage time, postoperative total drainage flow, postoperative complications, postoperative recurrence, tumor margin positive rate, hospital days, and cosmetic effect.

### 2.5 Quality assessment

Two autonomous reviewers evaluated the quality assessment in the trials that were included. We employed the Newcastle-Ottawa Scale (NOS) to evaluate the quality of retrospective literature in this study. In the event of any inconsistencies, the contested findings were resolved by engaging in collaborative deliberation.

### 2.6 Statistical analysis

The analyses were conducted using Stata 12.0. The continuous variables were compared using the weighted mean difference (WMD) and a 95% confidence interval (CI). The relative ratio (RR) was employed to compare binary variables, in conjunction with a 95% CI. The medians and interquartile ranges of continuous data were transformed into the mean and standard deviation. The statistical heterogeneity among the included studies was assessed using the Cochrane’s Q test and the I^2^ index. Given that the papers included in the study are obtained from public literature, it is generally more logical to opt for the random effect model as the initial choice. A p-value less than 0.05 was deemed statistically significant.

## 3 Results

### 3.1 Search results

**Figure 1:**
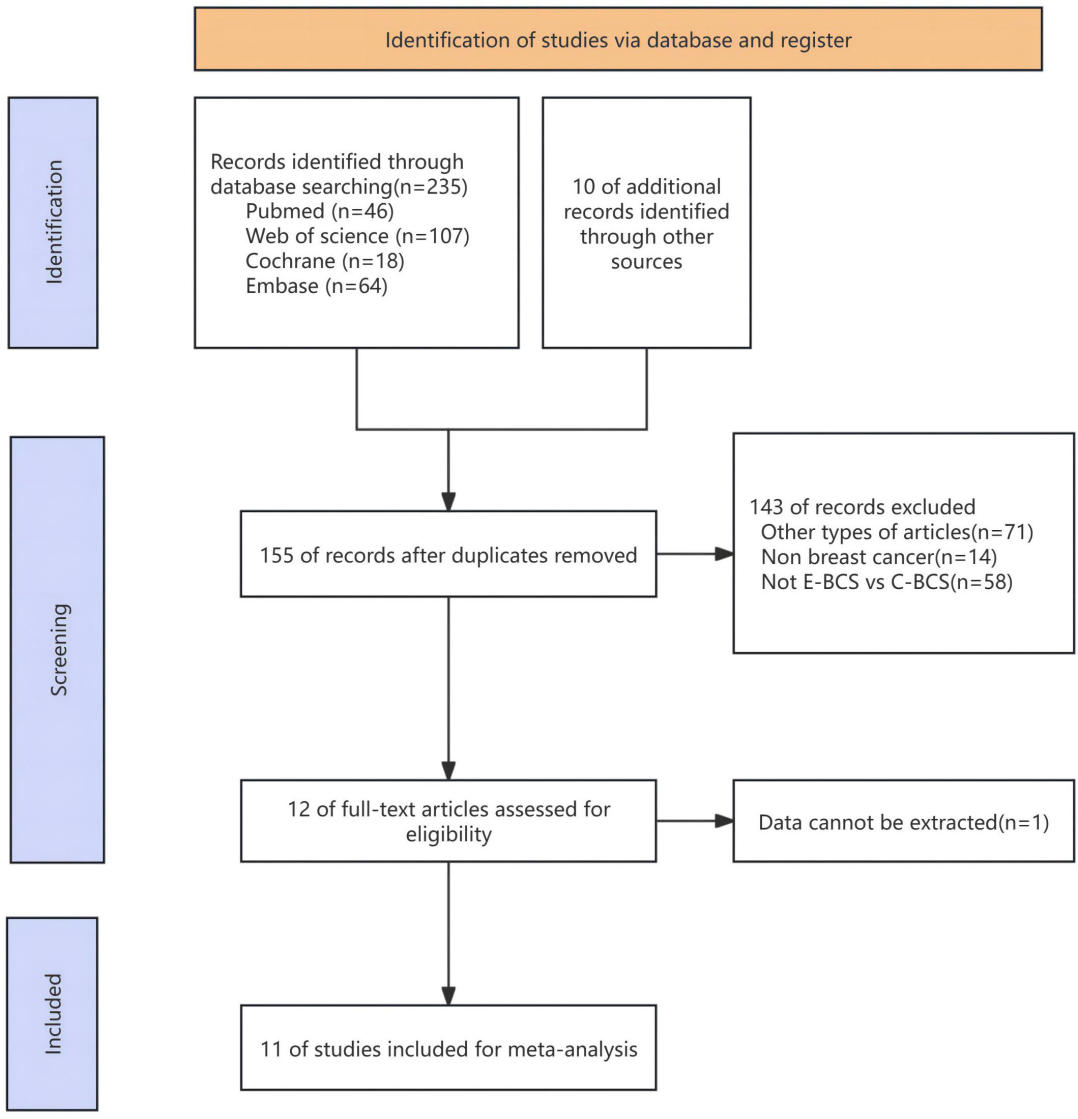
Flow chart of literature search strategies.

The process of selecting and incorporating articles was depicted in **Figure 1**. A total of 235 publications were obtained from four databases, and an additional two articles were discovered by reviewing the bibliographies of the mentioned papers. A total of 11 articles (27–37) were included in the final meta-analysis, following the established criteria for inclusion and exclusion. The procedure of selecting and including the research was depicted in **Figure 1**.

### 3.2 Study characteristics

The meta-analysis comprised a total of 11 studies, consisting of 3 prospective studies and 8 retrospective investigations. The meta-analysis comprised a total of 2562 individuals, including 852 patients in the E-BCS group and 1710 patients in the C-BCS group. The studies included were conducted in multiple countries, including of China (27, 28, 31, 35–37), Japan (30, 32–34), and Korea (29). The comprehensive data and fundamental attributes of the patients involved in the study are provided in **Table 1**.

**Table 1:** Characteristics of the included studies.

Study, year	country	design	study period	group	cases	Age (Mean ± SD)	TNM stage (0/I/II)
Fang Xie 2022 (27)	China	R	2017~2019	E	63	52.8 ± 8.7	0/32/32
				C	117	54.0 ± 10.4	0/49/68
Zi‐Han Wang 2018 (28)	China	R	2014~2015	E	35	50.8 ± 7.3	0/26/9
				C	35	51.0 ± 7.9	0/21/14
Hyung Seok Park 2011 (29)	Korea	R	2008~2010	E	40	51.1 ± 8.4	NA
				C	681	49.6 ± 9.5	NA
Hiroki Takahashi 2014 (30)	Japan	P	2009~2011	E	100	54.2 ± 10.7	4/61/35
				C	150	61.9 ± 14.3	12/74/64
Hung-wen Lai 2021 (31)	Taiwan	R	2011~2020	E	178	NA	NA
				C	24	NA	NA
Koji Yamashita 2006 (32)	Japan	P	2001~2005	E	80	53.7 ± 13.1	NA
				C	34	50.7 ± 13.0	NA
Nobuyuki Takemoto 2012 (33)	Japan	R	1997~2007	E	60	54.4 ± 12.5	5/42/13
				C	51	55.9 ± 11.4	5/36/10
Shinji Ozaki 2013 (34)	Japan	R	2005~2011	E	73	55.4 ± 10.0	14/36/23
				C	90	59.1 ± 12.1	11/43/36
Shou-Tung Chen 2021 (35)	Taiwan	R	2010~2020	E	149	NA	NA
				C	155	NA	NA
Hung-Wen Lai 2016 (36)	Taiwan	R	2009~2014	E	46	NA	NA
				C	322	NA	NA
Yinghui Liang 2020 (37)	China	P	2016~2018	E	28	43.39 ± 6.92	0/15/13
				C	51	48.46 ± 9.21	0/31/19

R, Retrospective study; P, Prospective study; E, Endoscopic Breast-conserving Surgery; C, Conventional Breast-conserving Surgery; NA, not available.

### 3.3 Quality assessment

The Newcastle-Ottawa Scale was utilized to evaluate the quality of studies included. Out of the 11 studies, 4 studies had a rating of 8 points while 7 studies received a rating of 7, suggesting that all of the included studies were of high quality. **Table 2** provides the detail of the quality assessment.

**Table 2:** Quality assessment of included studies.

Study, year	Selection	Comparability	Outcome	Total score
Fang Xie 2022 (27)	***	**	***	8
Zi‐Han Wang 2018 (28)	***	**	**	7
Hyung Seok Park 2011 (29)	****	*	**	7
Hiroki Takahashi 2014 (30)	****	**	*	7
Hung-wen Lai 2021 (31)	****	*	***	8
Koji Yamashita 2006 (32)	****	*	**	7
Nobuyuki Takemoto 2012 (33)	***	**	**	7
Shinji Ozaki 2013 (34)	****	**	**	8
Shou-Tung Chen 2021 (35)	***	**	***	8
Hung-Wen Lai 2016 (36)	****	*	**	7
Yinghui Liang 2020 (37)	****	*	**	7

### 3.4 Clinical outcomes

#### 3.4.1 Operation time (min)

**Figure 2:**
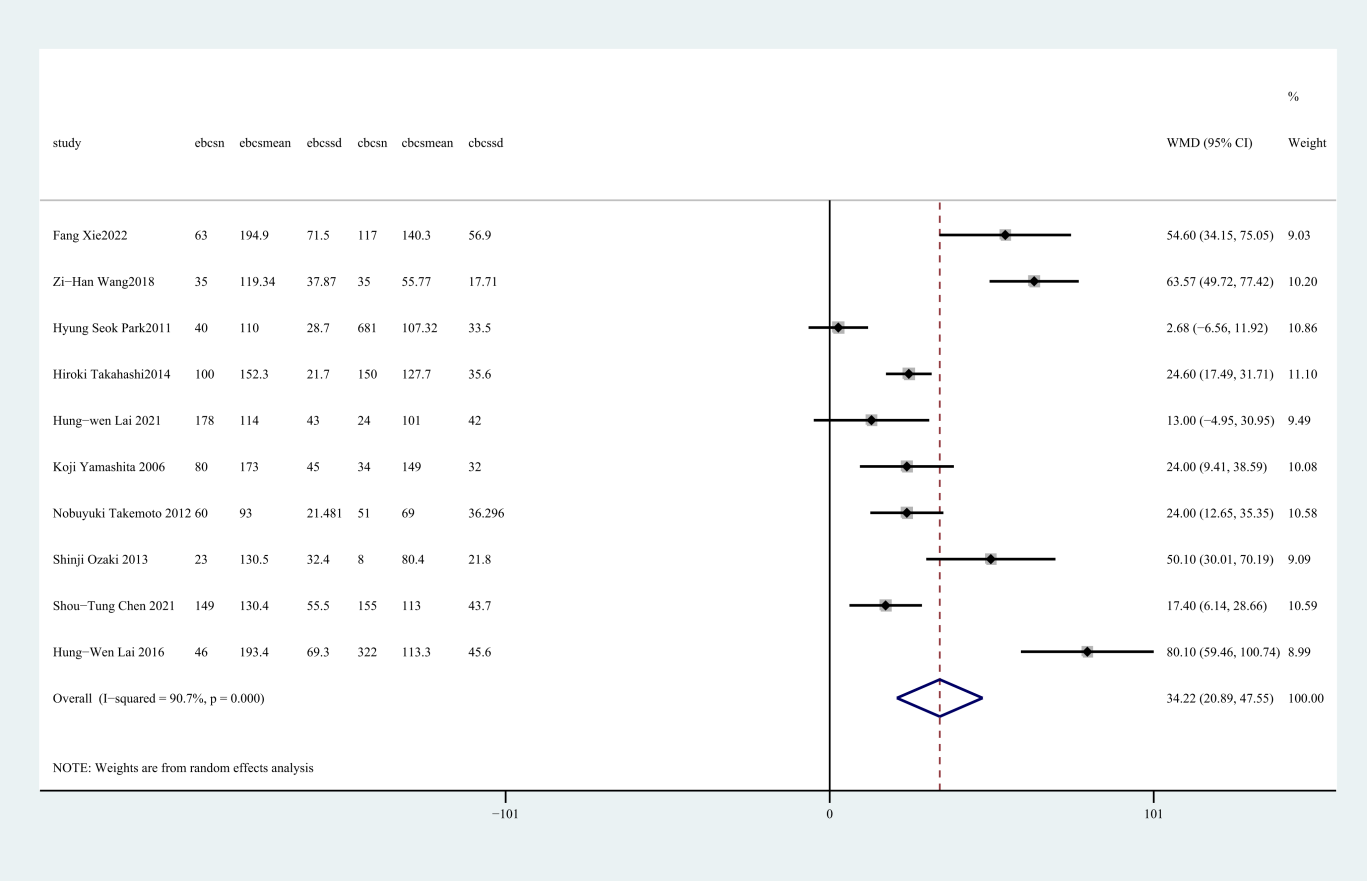
Forest plot of the meta-analysis for operation time.

Operation time was reported in ten studies (27–36). The pooled results showed that C-BCS had a significantly shorter operation time than E-BCS (WMD = 34.22, 95%CI: 20.89~47.55, P=0.000, I^2^ = 90.7%) (**Figure 2**).

#### 3.4.2 Blood loss (ml)

**Figure 3:**
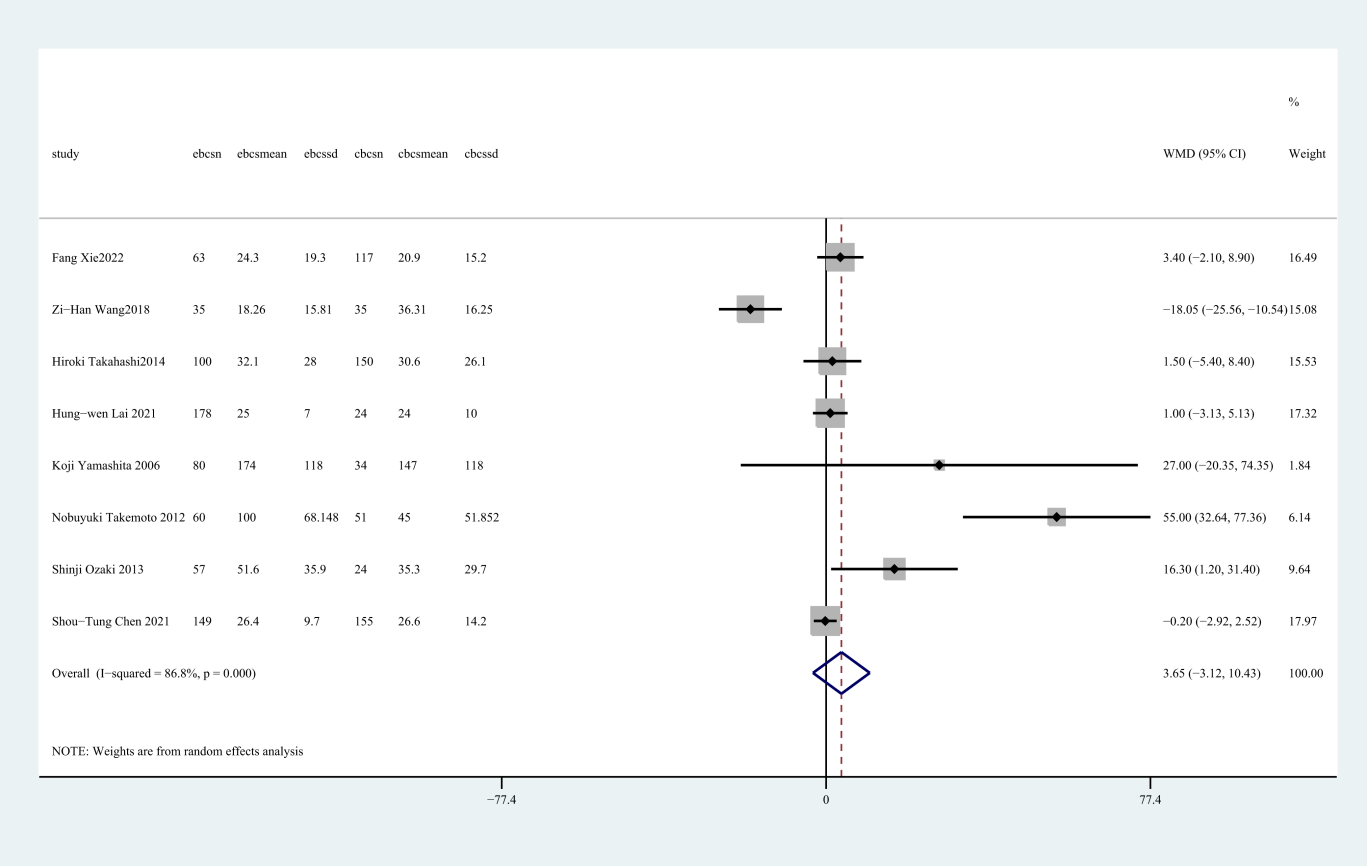
Forest plot of the meta-analysis for blood loss.

Eight studies (27, 28, 30–35) reported blood loss. There was no statistically significant distinction observed between the two groups in terms of blood loss (WMD = 3.65, 95%CI: -3.12 to 10.43, P=0.291, I^2^ = 86.8%) (**Figure 3**).

#### 3.4.3 Length of incision (cm)

**Figure 4:**
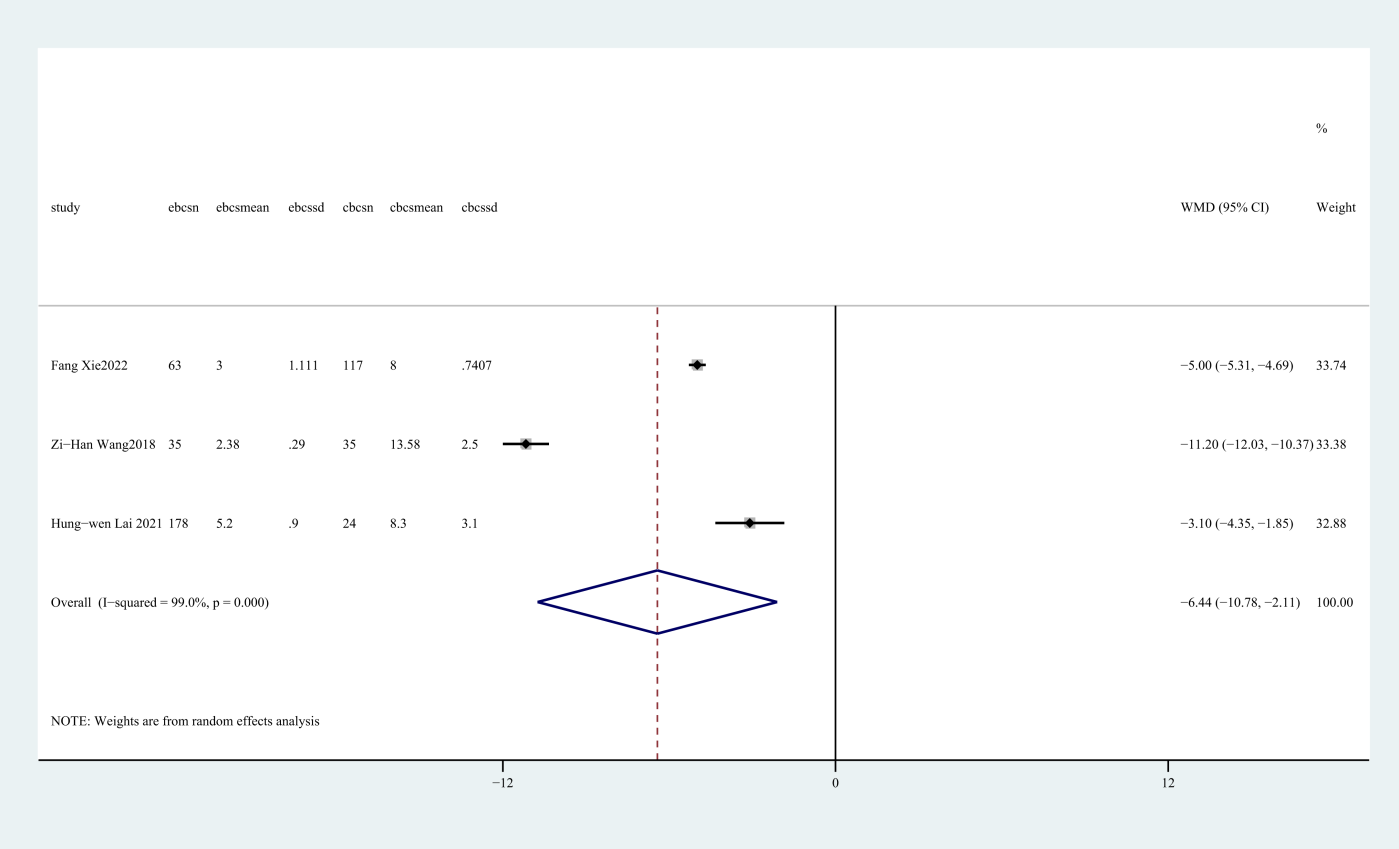
Forest plot of the meta-analysis for length of incision.

Length of incision was reported in three studies (27, 28, 31). The aggregated findings indicated a notable disparity between two groups, with E-BCS exhibiting a reduced incision length compared to C-BCS (WMD = -6.44, 95%CI: -10.78 to -2.11, P=0.004, I^2^ = 99.0%) (**Figure 4**).

#### 3.4.4 Drainage duration(day)

**Figure 5:**
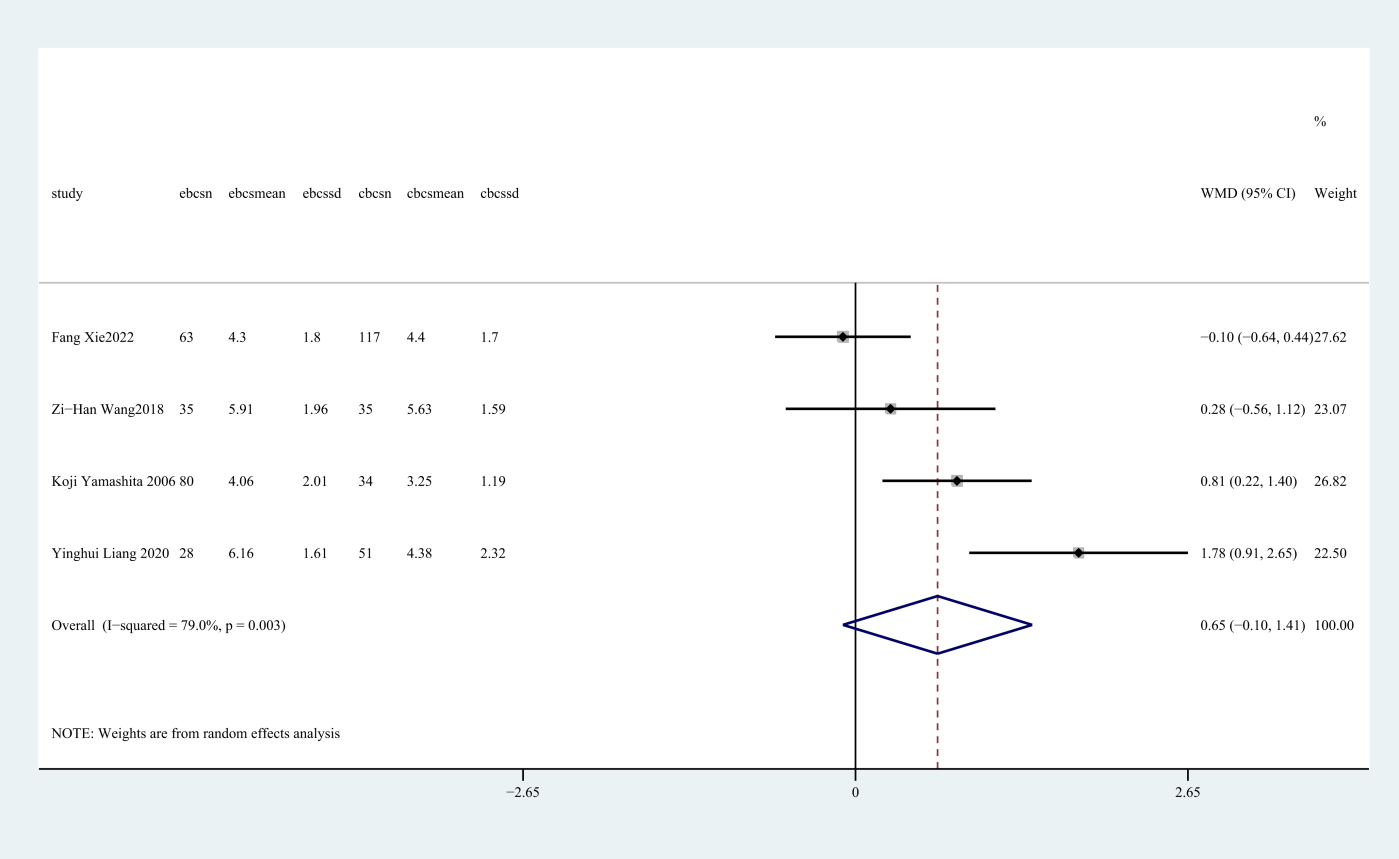
Forest plot of the meta-analysis for drainage duration.

Drainage duration was recorded in four studies (27, 28, 32, 37). There was no statistically significant disparity in the duration of drainage time between the two groups (WMD = 0.65, 95%CI: -0.10 to 1.41, P=0.089, I^2^ = 79.0%) (**Figure 5**).

#### 3.4.5 Total postoperative drainage volume (ml)

**Figure 6:**
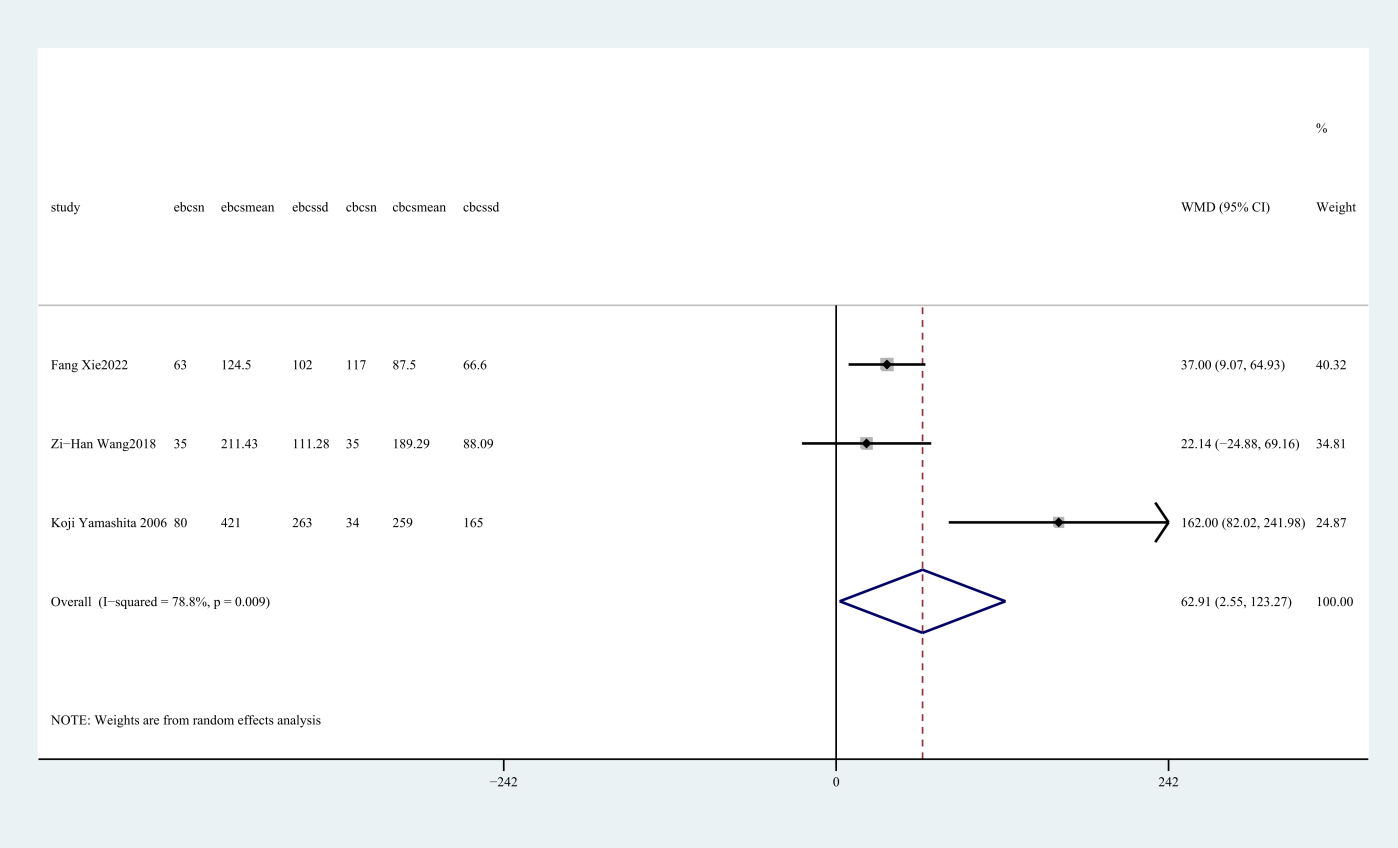
Forest plot of the meta-analysis for total drainage volume.

Three studies (27, 28, 32) reported total postoperative drainage volume. The pooled results showed that C-BCS had significantly lower total postoperative drainage volume than E-BCS (WMD = 62.9, 95%CI: 2.55~ 123.27, P=041, I^2^ = 78.8%) (**Figure 6**).

#### 3.4.6 Positive rate of tumor resection margins

**Figure 7:**
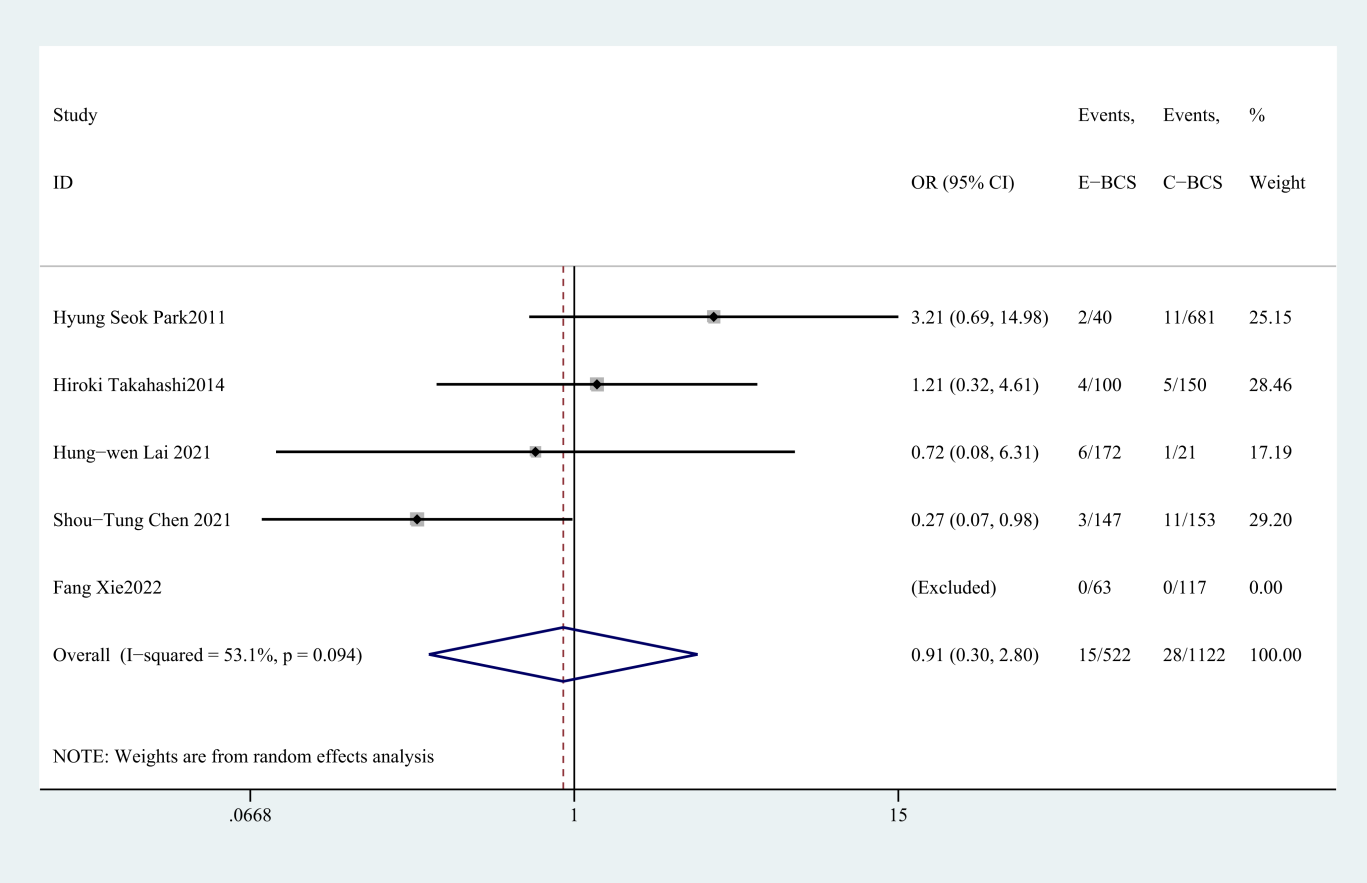
Forest plot of the meta-analysis for positive rate of tumor resection margins.

Five studies (27, 29–31, 35) recorded the positive rate of tumor margins. There was no statistically significant difference between E-BCS and C-BCS (OR = 0.91, 95%CI: 0.30 to 2.80, P=0.872, I^2^ = 53.1) (**Figure 7**).

#### 3.4.7 Recurrence rate

**Figure 8:**
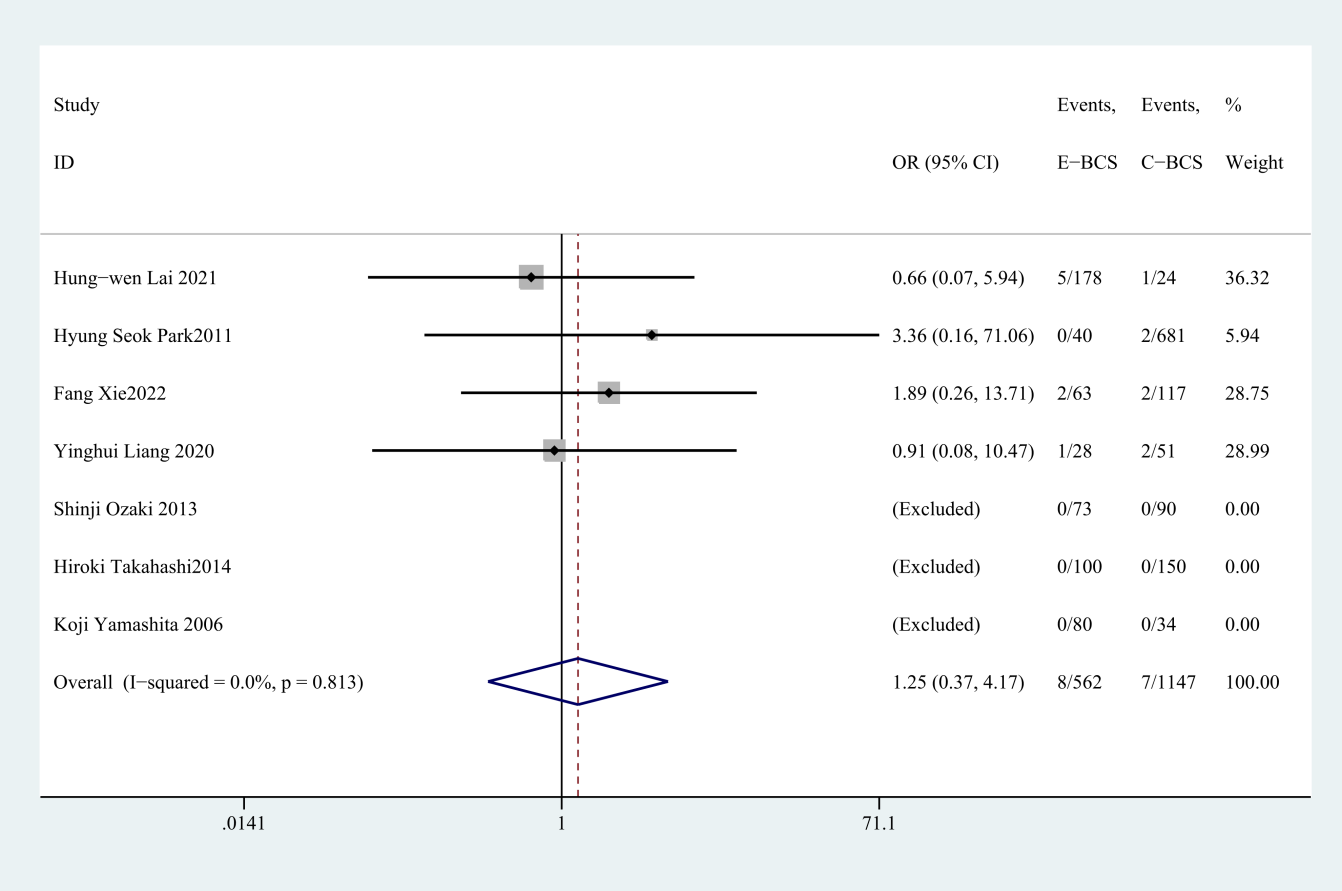
Forest plot of the meta-analysis for recurrence rate.

Seven studies (27, 29–32, 34, 37) reported postoperative recurrence rate. There was no statistically significant disparity in recurrence rate between E-BCS and C-BCS (OR = 1.25, 95%CI: 0.37 to 4.17, P=0.721, I^2^ = 0) (**Figure 8**).

#### 3.4.8 Complication rate

**Figure 9:**
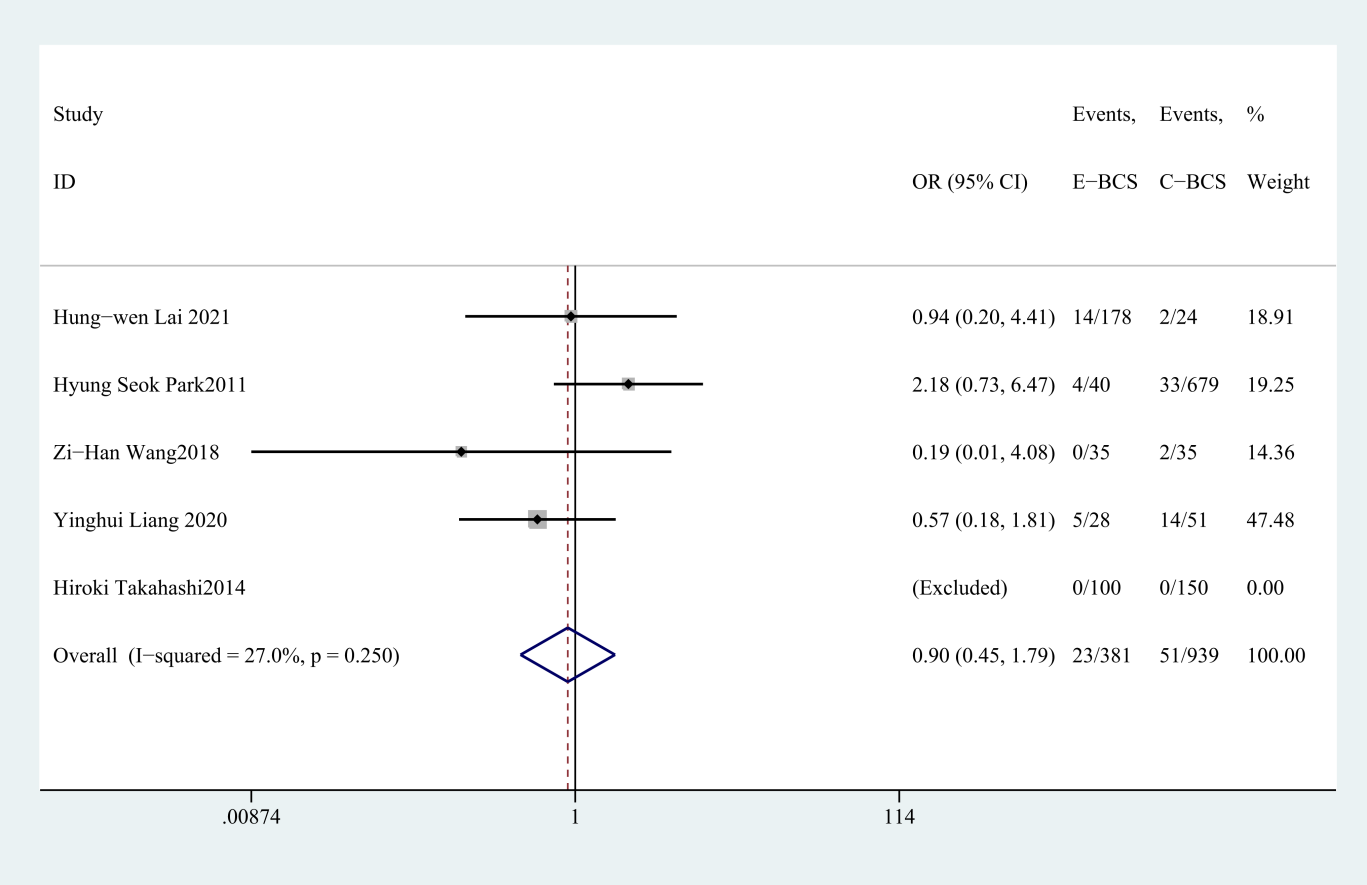
Forest plot of the meta-analysis for complication rate.

Five studies (28–31, 37)reported the rate of postoperative complications. The pooled results indicated that there was no statistically significant difference between two groups (OR = 0.9, 95%CI: 0.45 to 1.79, P=0.756, I^2^ = 27%) (**Figure 9**).

#### 3.4.9 Cosmetic score

**Figure 10:**
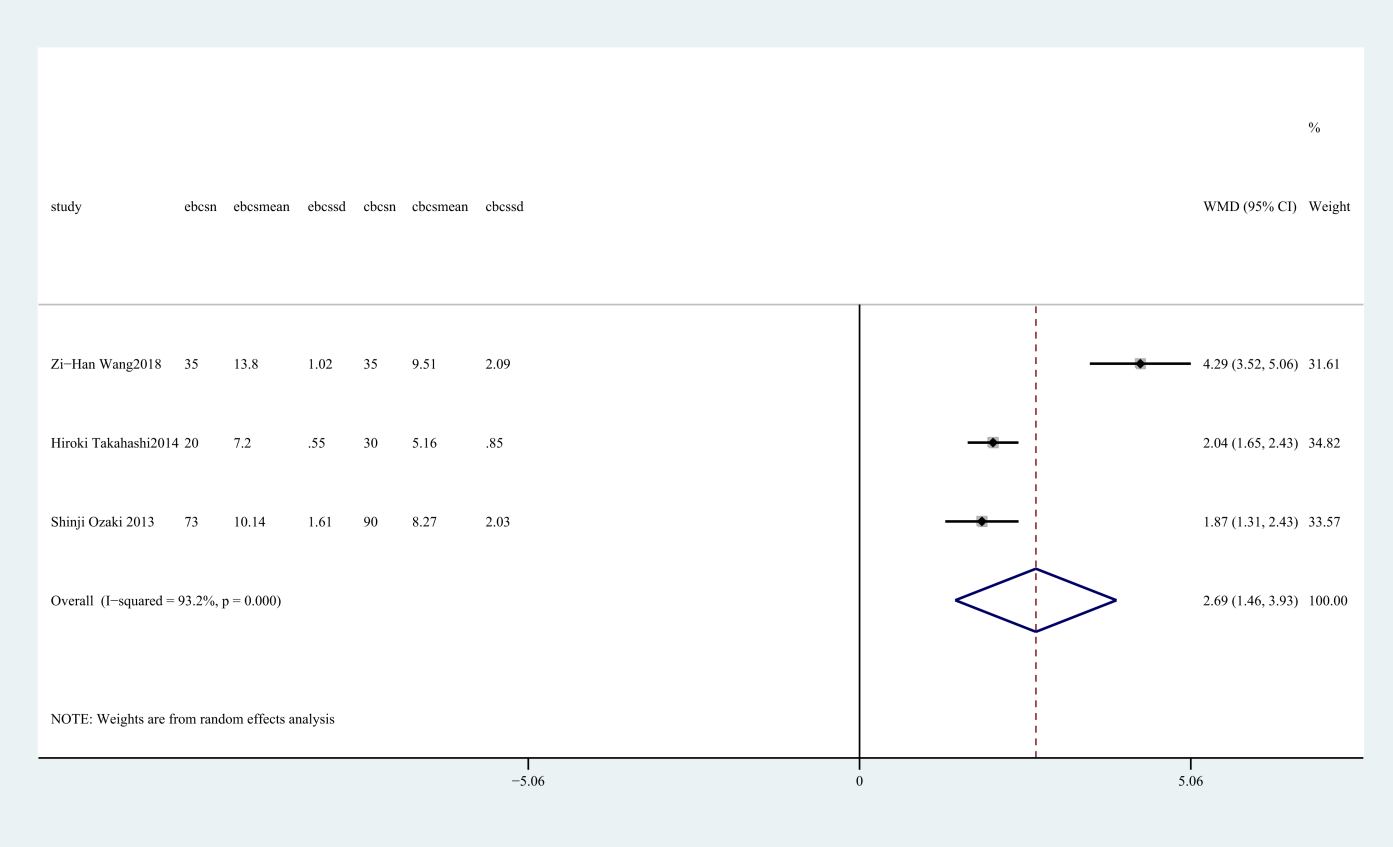
Forest plot of the meta-analysis for cosmetic score.

Three studies (28, 30, 34) reported cosmetic score. There was a notable disparity between two groups, with E-BCS exhibiting a superior cosmetic outcome compared to C-BCS (WMD = 2.69, 95%CI: 1.46 to 3.93, P=0.001, I^2^ = 93.2%) (**Figure 10**).

#### 3.4.10 Hospital days (day)

**Figure 11:**
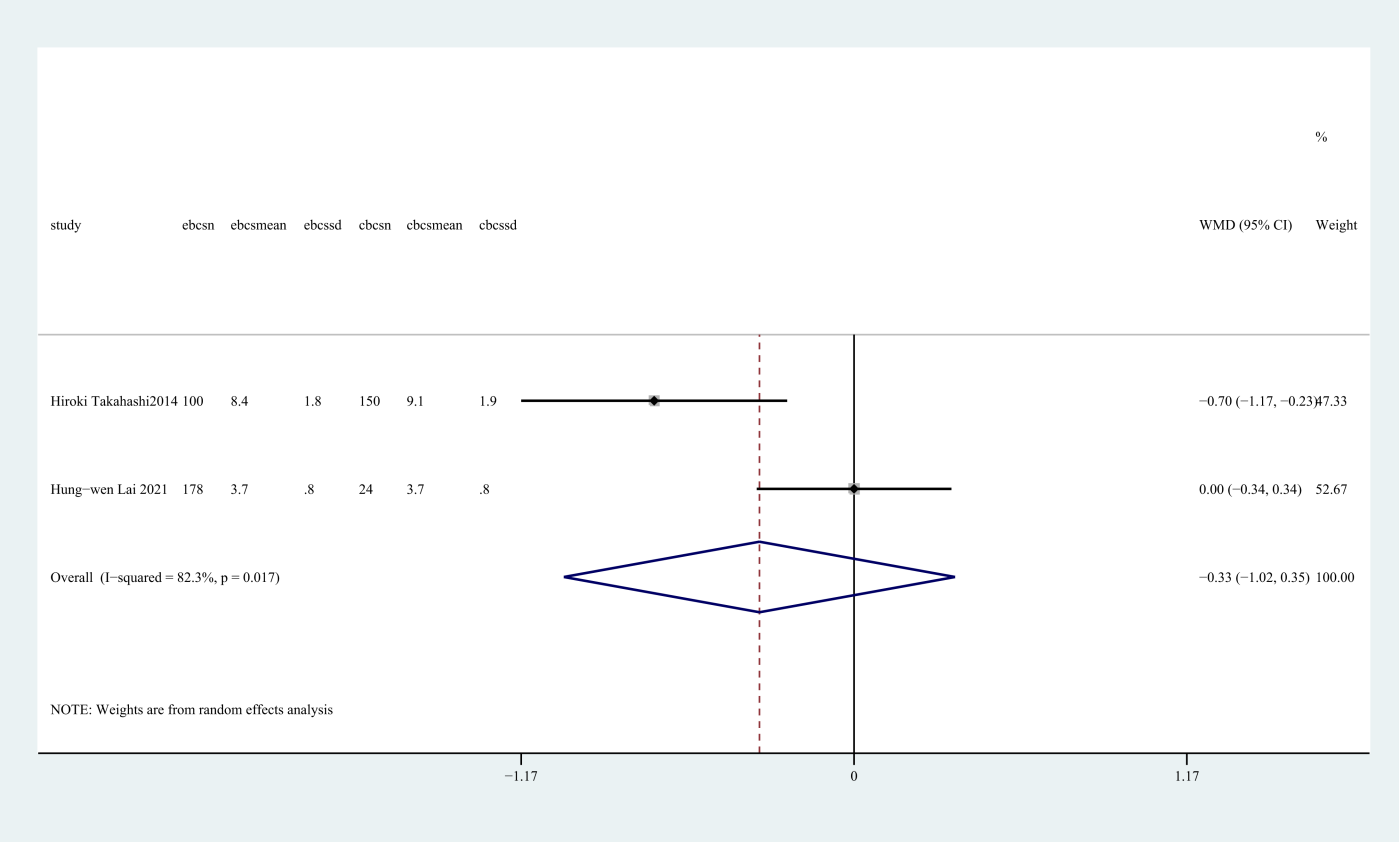
Forest plot of the meta-analysis for hospital days.

Two studies (30, 31) reported hospital days. There was no statistically significant difference between two groups regarding hospital days (WMD = -0.33, 95%CI: -1.02~0.35, P=0.343, I^2^ = 82.3%) (**Figure 11**).

#### 3.4.11 Metastasis rate

**Figure 12:**
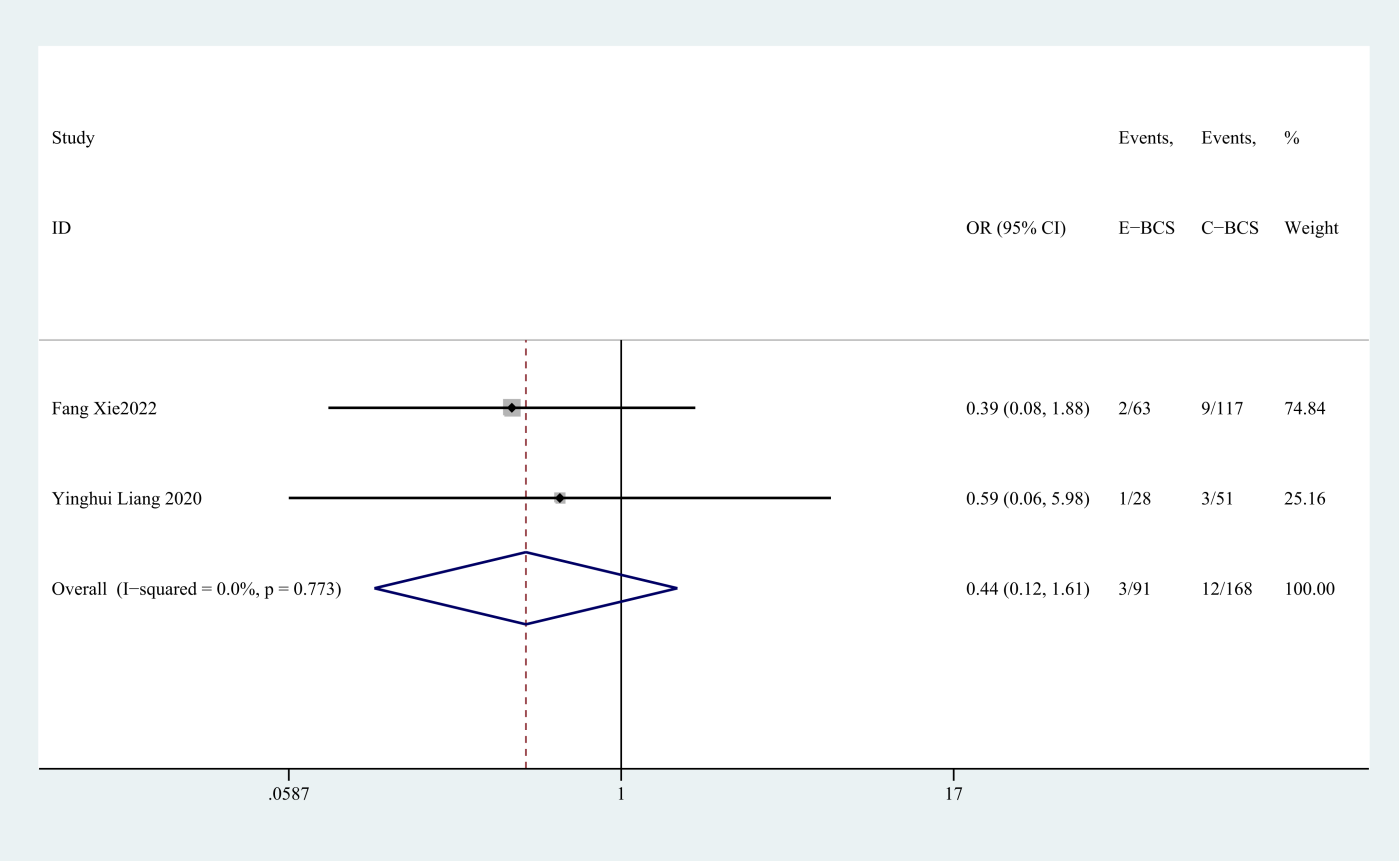
Forest plot of the meta-analysis for metastasis rate.

Two trials (27, 37) reported transfer rate. There was no statistically significant difference between two groups (OR = 0.44, 95% CI: 0.12 to 1.61, P=0.217, I^2^ = 0) (**Figure 12**).

### 3.5 Publication bias

**Figure 13:**
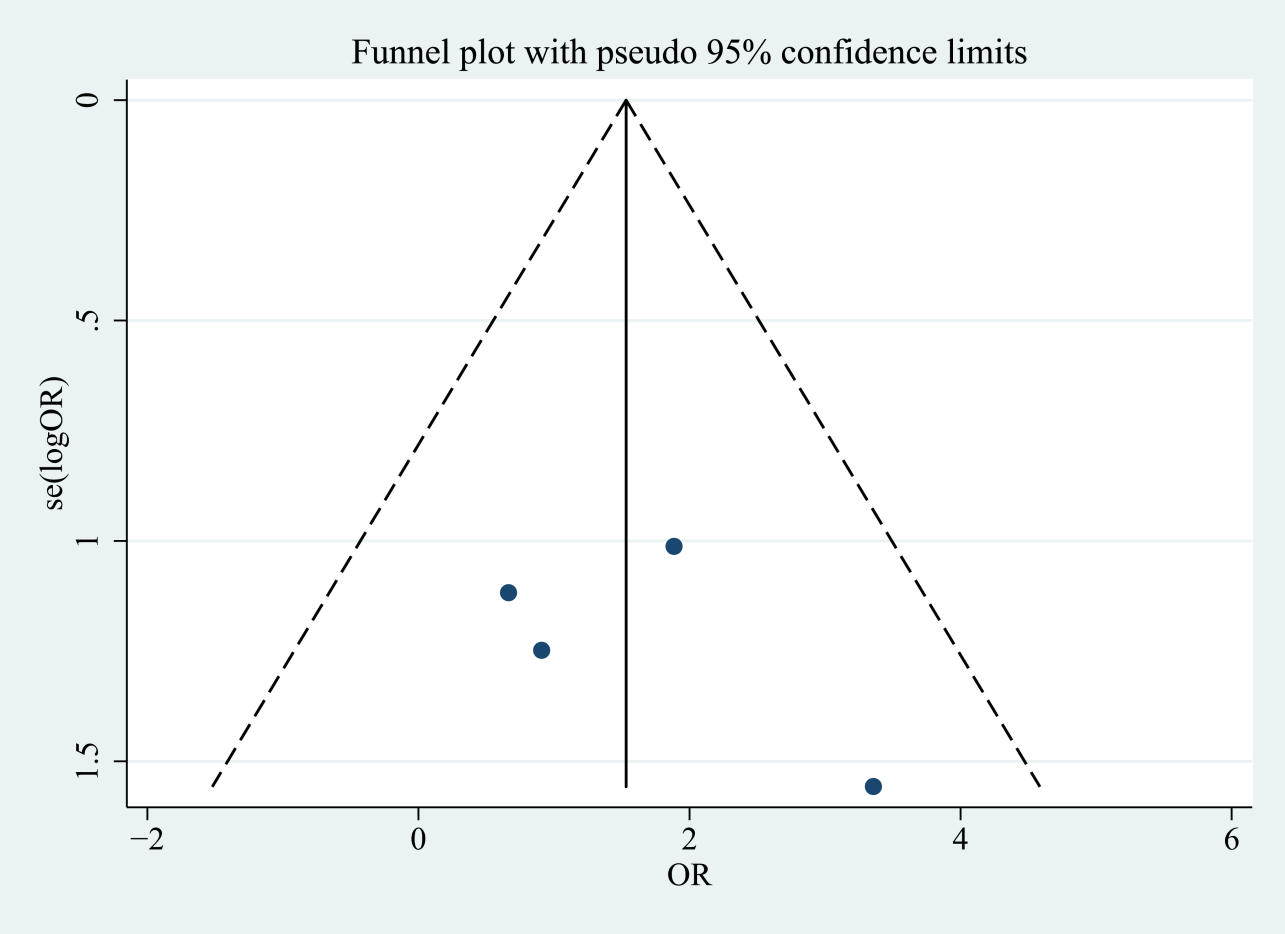
Funnel plot for recurrence rate.

A funnel plot was performed to evaluate publication bias in relation to the recurrence rate (**Figure 13**). The bilateral symmetric funnel plot of the recurrence rate did not provide any substantial indication of publication bias.

## 4 Discussion

BCS is the recommended treatment for women who have early-stage breast cancer (38–40). Nevertheless, C-BCS continues to have constraints in achieving satisfactory breast cosmetic results (8–10), so progressively falling short of meeting patient expectations (41). Endoscopy, a minimally invasive procedure, has been employed for over two decades (21, 22, 37, 42) in the management of breast cancer. The core principle involves utilizing discreet incisions in inconspicuous regions to enhance cosmetic outcomes (43). Endoscopic surgery has become increasingly prevalent in many surgical cases over the past decade and is now seen as a viable substitute for traditional open surgery. However, there are obstacles to endoscopic technology, and endoscopic breast surgery requires a series of specialist surgical procedures. The operation’s safety in regard to tumor treatment is not well-supported, and there is a chance of tumor diffusion (10). Therefore, the present meta-analysis was performed to compare the effectiveness and safety of E-BCS versus C-BCS in patients with early-stage breast cancer.

The oncological safety of E-BCS is frequently subject to scrutiny and skepticism due to doubts and worries regarding the effectiveness of resection performed through a small and inconspicuous incision (44). However, the findings of our study indicate that there was no statistically significant disparity between E-BCS and C-BCS in terms of the positive rate of tumor resection margins. Additionally, E-BCS did not exhibit a greater recurrence rate or metastasis rate when compared to C-BCS. In the E-BCS procedure, the surgeon benefited from carbon dioxide insufflation since it significantly expanded the available workspace for the surgical team, allowing them to execute the procedure with a single small incision (27). Short- and medium-term oncological outcomes are not compromised since it enables tumor excision with appropriate margins. Improved visualization with light handle retractors and enhanced precision for wide excision are further benefits (43).

In comparison to C-BCS, E-BCS reduces surgical incision length and yields better cosmetic outcomes, as shown in the present meta-analysis. Consistent findings have been reported in earlier studies (43, 45–47). Compared to C-BCS, E-BCS offers a number of benefits. Firstly, the SIE-BCS offers an aesthetic benefit due to the shorter incisions required compared to C-BCS. The technique of creating the single-port incision by following the natural axillary wrinkles further enhances this benefit, as it is then concealed by the upper limb and the axillary fossa (27). The second advantage of endoscopic surgery over direct vision surgery is the increased area that can be used to separate the pectoralis major muscle from its fascia. This, together with the broader skin flap that is created, enables suturing the surrounding dissected breast tissue more easier. This makes it easier for the space left behind after tissue removal to be adequately filled. Therefore, endoscopic surgery improves upon traditional breast-conserving surgery performed under direct vision in terms of cosmetic results by employing the aforementioned approaches (30).

Our findings indicated that E-BCS had a longer operation time than C-BCS. Several factors may contribute to it. To start, it’s a novel surgical method, so doctors who have never done endoscopic surgery before still have a lot to learn (37). Secondly, the surgical field of E-BCS is restricted and necessitates additional time for both preparation and execution of the procedure (43, 46, 47). Eun-Kyu Lee et al (25) found that, with the exception of the 3 instances that had axillary node dissection, the early 9 cases had a substantially longer operational time (178 minutes) compared to the latter 8 cases (130 minutes) (P < 0.001).The duration of endoscopic surgery would be reduced if the surgeon completes a period of learning. In another study (44), it was found that using the CUSUM approach for learning curve analysis, a total of 15 cases were required to achieve a considerable reduction in operation time. Specifically, the mean operation time decreased from 208 ± 53 minutes to 121 ± 37 minutes. Once the first learning curve was overcome, the operation time continued to decrease as more case experience was gained.

As far as we know, this meta-analysis has included the largest number of articles that compare the outcomes of E-BCS and C-BCS in treating early-stage breast cancer. This could lead to a more reliable conclusion. The results of our study offer useful insights into the clinical outcomes of surgical techniques that contribute to clinical practice and research in the field of breast cancer. Nevertheless, we recognize the potential limitations of our study. Initially, we included only 11 articles that met our inclusion criteria. The results obtained from these studies were unstable due to the limited sample size. Furthermore, the meta-analysis was compromised to some extent by the fact that all the studies included were non-randomized controlled trials, which diminished its trustworthiness. Furthermore, the limited duration of the included studies was inadequate to gather a satisfactory number of target events, such as the rate of recurrence and metastasis. This may have led to the results being unreliable. Failure to account for confounding factors, such as variations in countries, case inclusion criteria, medical equipment, and surgical techniques, can lead to research heterogeneity and bias. Hence, in order to provide additional validation on the safety and effectiveness of E-BCS, it is imperative to conduct more multicenter, randomized controlled trials with extended follow-up periods.

In conclusion, our research demonstrated that E-BCS was both feasible and safe for treating early-stage breast cancer. E-BCS offers clear benefits in terms of incision length and cosmetic outcome, without showing an increased risk of recurrence or metastasis.

## Data availability statement

The original contributions presented in the study are publicly available. This data can be found here: https://doi.org/10.6084/m9.figshare.25596849.v1.

## Author contributions

LL: Conceptualization, Formal analysis, Investigation, Writing – original draft. YL: Conceptualization, Formal analysis, Investigation, Writing – original draft. CL: Data curation, Supervision, Validation, Visualization, Writing – original draft. MH: Data curation, Supervision, Validation, Visualization, Writing – original draft. WL: Funding acquisition, Resources, Writing – review & editing. TQ: Funding acquisition, Resources, Writing – review & editing.
