# Supplementary material for: Comparison of endoscopic breast-conserving surgery versus conventional breast-conserving surgery for the treatment of early-stage breast cancer: a meta-analysis
Source: Front Oncol. 2024 Aug 6;14:1419123. doi: 10.3389/fonc.2024.1419123 (PMC11333215; doi:10.3389/fonc.2024.1419123)
Supplement: Supplementary file 1 [file Table_1.docx]

| pubmed | | |
| --- | --- | --- |
| No. | Query | Results |
| 1 | (((((((((((((((((((((((((((((((((((((Breast Neoplasms[Title/Abstract]) OR (Breast Neoplasm[Title/Abstract])) OR (Neoplasm, Breast[Title/Abstract])) OR (Breast Tumors[Title/Abstract])) OR (Breast Tumor[Title/Abstract])) OR (Tumor, Breast[Title/Abstract])) OR (Tumors, Breast[Title/Abstract])) OR (Neoplasms, Breast[Title/Abstract])) OR (Breast Cancer[Title/Abstract])) OR (Cancer, Breast[Title/Abstract])) OR (Mammary Cancer[Title/Abstract])) OR (Cancer, Mammary[Title/Abstract])) OR (Cancers, Mammary[Title/Abstract])) OR (Mammary Cancers[Title/Abstract])) OR (Malignant Neoplasm of Breast[Title/Abstract])) OR (Breast Malignant Neoplasm[Title/Abstract])) OR (Breast Malignant Neoplasms[Title/Abstract])) OR (Malignant Tumor of Breast[Title/Abstract])) OR (Breast Malignant Tumor[Title/Abstract])) OR (Breast Malignant Tumors[Title/Abstract])) OR (Cancer of Breast[Title/Abstract])) OR (Cancer of the Breast[Title/Abstract])) OR (Mammary Carcinoma, Human[Title/Abstract])) OR (Carcinoma, Human Mammary[Title/Abstract])) OR (Carcinomas, Human Mammary[Title/Abstract])) OR (Human Mammary Carcinomas[Title/Abstract])) OR (Mammary Carcinomas, Human[Title/Abstract])) OR (Human Mammary Carcinoma[Title/Abstract])) OR (Mammary Neoplasms, Human[Title/Abstract])) OR (Human Mammary Neoplasm[Title/Abstract])) OR (Human Mammary Neoplasms[Title/Abstract])) OR (Neoplasm, Human Mammary[Title/Abstract])) OR (Neoplasms, Human Mammary[Title/Abstract])) OR (Mammary Neoplasm, Human[Title/Abstract])) OR (Breast Carcinoma[Title/Abstract])) OR (Breast Carcinomas[Title/Abstract])) OR (Carcinoma, Breast[Title/Abstract])) OR (Carcinomas, Breast[Title/Abstract]) | 383919 |
| 2 | ((((((((endoscopic[Title/Abstract]) OR (endoscopy[Title/Abstract])) OR (laparoscopy[Title/Abstract])) OR (Laparoscopic[Title/Abstract])) OR (Mastoscopy[Title/Abstract])) OR (endoscopy-assisted[Title/Abstract])) OR (endoscope-assisted[Title/Abstract])) OR (endoscopic assisted[Title/Abstract])) OR (Minimally-invasive[Title/Abstract]) | 452070 |
| 3 | (((((breast-conserving surgery[Title/Abstract]) OR (BCS[Title/Abstract])) OR (breast-conserving therapy[Title/Abstract])) OR (breast sparing surgery[Title/Abstract])) OR (Conserving[Title/Abstract])) OR (sparing[Title/Abstract]) | 81671 |
| 4 | #1 AND#2 AND #3 | 199 |
| 5 | (((randomized controlled trial [pt] OR controlled clinical trial [pt] OR randomized [tiab] OR placebo [tiab] OR clinical trials as topic [mh: noexp] OR randomly [tiab] OR trial [ti]) NOT (animals [mh] NOT humans [mh])) OR (Prospective[Title/Abstract])) OR (Retrospective[Title/Abstract]) | 2837607 |
| 6 | #4 AND #5 | 46 |

| Cochrane library | | |
| --- | --- | --- |
| No. | Query | Results |
| 1 | (Breast Neoplasms OR Breast Neoplasm OR Neoplasm, Breast OR Breast Tumors OR Breast Tumor OR Tumor, Breast OR Tumors, Breast OR Neoplasms, Breast OR Breast Cancer OR Cancer, Breast OR Mammary Cancer OR Cancer, Mammary OR Cancers, Mammary OR Mammary Cancers OR Malignant Neoplasm of Breast OR Breast Malignant Neoplasm OR Breast Malignant Neoplasms OR Malignant Tumor of Breast OR Breast Malignant Tumor OR Breast Malignant Tumors OR Cancer of Breast OR Cancer of the Breast OR Mammary Carcinoma, Human OR Carcinoma, Human Mammary OR Carcinomas, Human Mammary OR Human Mammary Carcinomas OR Mammary Carcinomas, Human OR Human Mammary Carcinoma OR Mammary Neoplasms, Human OR Human Mammary Neoplasm OR Human Mammary Neoplasms OR Neoplasm, Human Mammary OR Neoplasms, Human Mammary OR Mammary Neoplasm, Human OR Breast Carcinoma OR Breast Carcinomas OR Carcinoma, Breast OR Carcinomas, Breast):ab,ti,kw | 46763 |
| 2 | (Endoscopic OR endoscopy OR laparoscopy OR Laparoscopic OR Mastoscopy OR endoscopy-assisted OR endoscope-assisted OR endoscopic assisted OR Minimally-invasive):ab,ti,kw | 62308 |
| 3 | (breast-conserving surgery OR BCS OR breast-conserving therapy OR breast sparing surgery OR Conserving OR sparing):ab,ti,kw | 9063 |
| 4 | #1 AND#2 AND #3 | 19 |
| 5 | (randomized controlled trial OR controlled clinical trial OR Randomized OR placebo OR clinical trials as topic OR randomly OR Trial OR Prospective OR Retrospective):ti,ab,kw | 1513294 |
| 6 | #4 AND #5 | 18 |

| Embase | | |
| --- | --- | --- |
| No. | Query | Results |
| 1 | 'breast neoplasms':ab,ti OR 'breast neoplasm':ab,ti OR 'neoplasm, breast':ab,ti OR 'breast tumors':ab,ti OR 'breast tumor':ab,ti OR 'tumor, breast':ab,ti OR 'tumors, breast':ab,ti OR 'neoplasms, breast':ab,ti OR 'breast cancer':ab,ti OR 'cancer, breast':ab,ti OR 'mammary cancer':ab,ti OR 'cancer, mammary':ab,ti OR 'cancers, mammary':ab,ti OR 'mammary cancers':ab,ti OR 'malignant neoplasm of breast':ab,ti OR 'breast malignant neoplasm':ab,ti OR 'breast malignant neoplasms':ab,ti OR 'malignant tumor of breast':ab,ti OR 'breast malignant tumor':ab,ti OR 'breast malignant tumors':ab,ti OR 'cancer of breast':ab,ti OR 'cancer of the breast':ab,ti OR 'mammary carcinoma, human':ab,ti OR 'carcinoma, human mammary':ab,ti OR 'carcinomas, human mammary':ab,ti OR 'human mammary carcinomas':ab,ti OR 'mammary carcinomas, human':ab,ti OR 'human mammary carcinoma':ab,ti OR 'mammary neoplasms, human':ab,ti OR 'human mammary neoplasm':ab,ti OR 'human mammary neoplasms':ab,ti OR 'neoplasm, human mammary':ab,ti OR 'neoplasms, human mammary':ab,ti OR 'mammary neoplasm, human':ab,ti OR 'breast carcinoma':ab,ti OR 'breast carcinomas':ab,ti OR 'carcinoma, breast':ab,ti OR 'carcinomas, breast':ab,ti | 538552 |
| 2 | 'endoscopic':ab,ti OR 'endoscopy':ab,ti OR 'laparoscopy':ab,ti OR 'laparoscopic':ab,ti OR 'mastoscopy':ab,ti OR 'endoscopy-assisted':ab,ti OR 'endoscope-assisted':ab,ti OR 'endoscopic assisted':ab,ti OR 'minimally-invasive':ab,ti | 703815 |
| 3 | 'breast-conserving surgery':ab,ti OR 'bcs':ab,ti OR 'breast-conserving therapy':ab,ti OR 'breast sparing surgery':ab,ti OR 'conserving':ab,ti OR 'sparing':ab,ti | 124574 |
| 4 | #1AND#2 AND #3 | 283 |
| 5 | 'randomized controlled trial':ab,ti OR 'controlled clinical trial':ab,ti OR randomized:ab,ti OR placebo:ab,ti OR 'clinical trials as topic':ab,ti OR randomly:ab,ti OR trial:ab,ti OR prospective:ab,ti OR retrospective:ab,ti | 4215786 |
| 6 | #4 AND #5 | 64 |

| Web of science | | |
| --- | --- | --- |
| No. | Query | Results |
| 1 | (((((((((((((((((((((((((((((((((((((TS=(Breast Neoplasms)) OR TS=(Breast Neoplasm)) OR TS=(Neoplasm, Breast)) OR TS=(Breast Tumors)) OR TS=(Breast Tumor)) OR TS=(Tumor, Breast)) OR TS=(Tumors, Breast)) OR TS=(Neoplasms, Breast)) OR TS=(Breast Cancer)) OR TS=(Cancer, Breast)) OR TS=(Mammary Cancer)) OR TS=(Cancer, Mammary)) OR TS=(Cancers, Mammary)) OR TS=(Mammary Cancers)) OR TS=(Malignant Neoplasm of Breast)) OR TS=(Breast Malignant Neoplasm)) OR TS=(Breast Malignant Neoplasms)) OR TS=(Malignant Tumor of Breast)) OR TS=(Breast Malignant Tumor)) OR TS=(Breast Malignant Tumors)) OR TS=(Cancer of Breast)) OR TS=(Cancer of the Breast)) OR TS=(Mammary Carcinoma, Human)) OR TS=(Carcinoma, Human Mammary)) OR TS=(Carcinomas, Human Mammary)) OR TS=(Human Mammary Carcinomas)) OR TS=(Mammary Carcinomas, Human)) OR TS=(Human Mammary Carcinoma)) OR TS=(Mammary Neoplasms, Human)) OR TS=(Human Mammary Neoplasm)) OR TS=(Human Mammary Neoplasms)) OR TS=(Neoplasm, Human Mammary)) OR TS=(Neoplasms, Human Mammary)) OR TS=(Mammary Neoplasm, Human)) OR TS=(Breast Carcinoma)) OR TS=(Breast Carcinomas)) OR TS=(Carcinoma, Breast)) OR TS=(Carcinomas, Breast) | 729588 |
| 2 | ((((((((TS=(endoscopic)) OR TS=(endoscopy)) OR TS=(laparoscopy)) OR TS=( Laparoscopic)) OR TS=(Mastoscopy)) OR TS=(endoscopy-assisted)) OR TS=(endoscope-assisted)) OR TS=(endoscopic assisted)) OR TS=(Minimally-invasive) | 492614 |
| 3 | (((((TS=(breast-conserving surgery)) OR TS=(BCS)) OR TS=(breast-conserving therapy)) OR TS=(breast sparing surgery)) OR TS=(Conserving)) OR TS=(sparing) | 480590 |
| 4 | #1AND#2 AND #3 | 336 |
| 5 | ((((((((TS=(randomized controlled trial )) OR TS=(controlled clinical trial )) OR TS=(Randomized)) OR TS=(placebo )) OR TS=(clinical trials as topic )) OR TS=(randomly )) OR TS=(Trial)) OR TS=(Prospective )) OR TS=(Retrospective) | 3924604 |
| 6 | #4 AND #5 | 107 |
